# Potential additional effects of iron chelators on antimicrobial- impregnated central venous catheters

**DOI:** 10.3389/fmicb.2023.1210747

**Published:** 2023-08-07

**Authors:** Kazuhiro Itoh, Hiroshi Tsutani, Yasuhiko Mitsuke, Hiromichi Iwasaki

**Affiliations:** ^1^Department of Internal Medicine, National Hospital Organization Awara Hospital, Awara, Japan; ^2^Division of Infection Control and Prevention, University of Fukui Hospital, Fukui, Japan

**Keywords:** central venous catheterization, catheter-related infections, catheter-related bacteremia, iron-chelating agents, biofilms

## Introduction

Central venous catheters (CVCs) play a clinically important role in various treatments, including venous pressure monitoring, the infusion of drugs, such as anti-cancer chemotherapy and antibiotics, parenteral nutrition, and blood transfusion (Saugel et al., 2017). However, compared with peripheral venous catheters, patients with indwelling CVCs are at increased risk of thrombosis, embolism, and infection (Ball and Singh, 2023). Catheter-related infections (CRIs) are thought to be associated with the formation of bacterial and fungal colonies on the catheter (Hanna et al., 2004). Microorganisms adhere to device surfaces, produce an extracellular polymeric matrix, and form biofilms. The biofilm-forming microorganisms then become a source of medical device-associated infection (Donlan, 2002). CRI is defined as a positive catheter tip culture and systemic inflammatory findings, while catheter-related bloodstream infection (CRBSI) is defined as CRI-criteria plus detection of the same organisms as in the catheter tip culture in peripheral blood cultures (Garner et al., 1988; Rockholt et al., 2023). CRBSIs have a reported mortality rate of 12%−40% depending on a variety of factors, such as comorbidities, the type of CVC used, and the type of pathogen, and are associated with longer hospital stays and increased medical costs (O'Grady et al., 2011; Böll et al., 2021; Ball and Singh, 2023).

Several preventive measures exist for CRIs, such as thorough hand hygiene, sterilization of the CVC site, and avoidance of catheter insertion at the femoral site. It is widely accepted that CVCs, which are foreign substances that can be a source of infection, should be removed as soon as possible. However, in clinical practice, situations arise in which long-term indwelling is required. Therefore, in such special situations, the Centers for Disease Control and Prevention (CDC) recommends the use of antimicrobial agents or antiseptic-impregnated catheters with comprehensive and adequate infection control measures, as shown Supplementary Figure S1 (O'Grady et al., 2011). Antimicrobial-impregnated catheters are a promising option for cases that require long-term CVC use (Böll et al., 2021). The purpose of this article was to summarize previous attempts to use antimicrobial/antiseptic impregnated catheters and provide a new perspective.

## Antimicrobial/antiseptic-impregnated catheters

The control of CRI remains a major challenge to overcome; hence, a multifaceted attempt should be conducted. Certainly, antimicrobial-impregnated catheters have achieved some success to date, but no established clinical efficacy has been achieved with antiseptic substances (*e.g*., silver), which have been studied for many years (Viola et al., 2017). One such catheter is the chlorhexidine/silver sulfadiazine catheter, but most studies have found that its effect on reducing CRBSIs is not significant (Viola et al., 2017). Minocycline/rifampicin and miconazole/rifampicin catheters have also been shown to reduce the incidence of CRBSIs in several studies, where the incidence of antibiotic resistance, which remains a concern, was low (Viola et al., 2017; Reitzel et al., 2020; Böll et al., 2021). In clinical trials of patients with cancer, minocycline/rifampicin-impregnated catheters have also been shown to reduce the incidence of CRBSIs when used for longer than 2 months (Hanna et al., 2004). The combination of chlorhexidine with minocycline and rifampicin on catheters has been reported to have enhanced antimicrobial activity (Raad et al., 2012). Chlorhexidine acts on cell membranes, minocycline on protein synthesis, and rifampin on RNA synthesis. Thus, the mechanism of action of the combination of these three drugs may be additive. However, the confirmed enhancement of activity is only the result of *in vitro* studies, and the possibility of clinical application needs to be confirmed in clinical trials (Viola et al., 2017). As discussed above, a combined approach with different mechanisms of action may enhance antimicrobial activity and broaden the spectrum. Therefore, new approaches that are not antimicrobial agents or antiseptic substances may produce additional benefits.

## Hypothesis

From a different perspective, attempts have been made to inhibit iron, which is essential for the growth and proliferation of pathogenic microorganisms, as a strategy to increase antimicrobial activity (Schwarz et al., 2019; Scott et al., 2020). Specifically, the strategy is to use iron chelators in combination with antibacterial and antifungal agents. We hypothesize that the impregnation of catheters with antimicrobial agents and iron chelators would increase their antimicrobial and anti-biofilm activity and inhibit microbial colony and biofilm formation, resulting in reduced CRI incidence and longer catheter use, as shown in Supplementary Figure S2. The surface and lumen of the catheter are impregnated with antimicrobial and iron-chelating agents, which are assumed to inhibit bacterial and fungal colony and biofilm formation by exerting antimicrobial and iron-chelating activities on the bacteria and fungi that attach to the surface.

## Support for the hypothesis

### Antimicrobial activity of iron chelators

Because of its redox potential, iron is involved in many biochemical reactions. Iron is an essential element for all living organisms, including bacterial pathogens (Dev and Babitt, 2017). It also plays an important role as a cofactor in various intracellular pathways, such as DNA synthesis and repair, electron transfer, oxygen transport, and immune response (Scott et al., 2020). Iron levels in the human body are strictly regulated to protect against the toxic effects of free iron, which catalyzes the generation of reactive oxygen species (ROS), and the utilization of iron by invading microorganisms (Dixon and Stockwell, 2014). Through nutritional immunity, humans inhibit microbial growth by starving microorganisms of the metal ions they need (Hood and Skaar, 2012; Palmer and Skaar, 2016). Bacteria and fungi bind iron using high-affinity chelating compounds, called siderophores, as a strategy to acquire iron. Numerous types of siderophores have been reported. Siderophores are categorized into three main structural families—carboxylates, catecholates, and hydroxamates—with the catecholate siderophore, enterobactin, showing the highest affinity for iron, even higher than that for the host iron-binding protein, transferrin (Ellermann and Arthur, 2017). Iron chelators may be useful in the treatment of infectious diseases by limiting the availability of iron, thereby inhibiting the production of ROS, and by inhibiting microbial growth due to nutrient restriction (Lehmann et al., 2021; Paterson et al., 2022).

To date, the combinations of vancomycin and deferasirox against methicillin-resistant *Staphylococcus aureus* (Luo et al., 2014), doxycycline, and the iron chelator CP762 against *Pseudomonas aeruginosa* (Faure et al., 2021), and the triple combination of doxycycline, deferasirox, and thiostrepton (an antibiotic against gram-positive bacteria) against *P. aeruginosa* and *Acinetobacter baumannii* (Chan et al., 2020) have shown promising effects both *in vitro* and in animal models. For fungi, animal model experiments of combination therapy with liposomal amphotericin B (L-AMB), micafungin, and deferasirox against *Aspergillus* and *Mucor* (Ibrahim et al., 2011), animal experiments of combination therapy with L-AMB and deferasirox against *Aspergillus fumigatus* (Ibrahim et al., 2010), and *in vitro* experiments of combination therapy with AMB and deferoxamine against *Cryptococcus* spp. (Chayakulkeeree et al., 2020) have indicated that these combinations are useful. Despite promising experimental data for the combination of deferasirox and L-AMB for mucormycosis (Ibrahim et al., 2007, 2011; Schwarz et al., 2019), unexpectedly, a higher mortality rate at 90 days was seen with the combination than with L-AMB alone in a randomized controlled trial (Spellberg et al., 2012). Patients were enrolled at multiple centers with heterogeneous patient populations, resulting in an imbalance in underlying diseases and risk factors, with more patients in the deferasirox group having hematologic malignancies, neutropenia, and pulmonary infections. This imbalance in the patient's background may have influenced the patient's prognosis (Spellberg et al., 2012). Therefore, clinical trials are inconclusive regarding the efficacy of iron chelation assisted therapy.

Nevertheless, iron-chelation therapy still has potential for clinical applications. Different types of iron-chelating agents have been shown to have different antimicrobial activities. Of those tested, a water-soluble agent containing iron-selective copolymers (denying iron to bacterial infections; DIBI) was the most effective (Iron Binding Polymers | Fe Pharma, 2021; Lehmann et al., 2021). Recent studies have reported the promising finding that a combination of DIBI and antimicrobials may overcome drug-resistant bacteria and fungi. DIBI in combination with ciprofloxacin has been shown to be successful in treating ciprofloxacin-resistant *A. baumannii*-infected mice (Parquet et al., 2019). Moreover, the combination of DIBI and fluconazole was found to inhibit the growth of fluconazole-resistant *Candida albicans* in an *in vitro* study (Savage et al., 2018). These findings suggest that the appropriate selection of a type of iron chelator and its combination with an antimicrobial agent may be useful for dealing with a broad spectrum of infectious pathogens, and may overcome antibiotic resistance mechanisms.

### Anti-biofilm activity of iron chelators

One notable problem related to CVC implantation is biofilm formation. Here, we further explain the relationship between iron chelators and biofilm formation. It has already been mentioned that iron is essential for bacterial and fungal growth and proliferation, but it is also required for biofilm formation (Nazik et al., 2015; Coraça-Huber et al., 2018; Firoz et al., 2021). Microorganisms produce biofilms that protect them against antimicrobials, making them drug resistant (Høiby et al., 2010; Singh et al., 2010; Brauner et al., 2016; Thi et al., 2020). Hence, methods are needed to inhibit the formation of biofilms and attack the microorganisms that have formed within them. Iron-chelation therapy has been tested in *P. aeruginosa* to explore adjunctive therapy for chronic *P. aeruginosa* infection. Deferiprone, a synthetic iron chelator, has been shown to inhibit biofilm formation (Houshmandyar et al., 2021). Furthermore, the combination of iron chelators and antimicrobial agents has shown enhanced biofilm inhibitory activity; examples include combination therapy with the iron (VI) chelator N, N'-bis (2-hydroxybenzyl) ethylenediamine-N, N'-diacetic acid and colistin in an *in vitro* experiment (Mettrick et al., 2020), and with the iron chelators deferoxamine or deferasirox and tobramycin (Moreau-Marquis et al., 2009). Lactoferrin, a component of human secretions, also has iron-chelating activity (Singh, 2004), and ALX-109, an investigational agent containing lactoferrin and hypothiocyanite (a bactericidal agent), has shown activity against *P. aeruginosa* biofilms in combination with tobramycin and aztreonam (Moreau-Marquis et al., 2015). Deferasirox has shown biofilm inhibitory effects on the periodontal bacterium *Prevotella intermedia* (Moon et al., 2013), and lactoferrin treatment has significantly reduced proinflammatory cytokines production by gingival fibroblasts infected with *P. intermedia*. Moreover, an observational clinical trial of patients with periodontitis showed that treatment with lactoferrin reduced proinflammatory cytokines such as interleukin 6, edema, bleeding, pocket depth, and gingival and plaque indices in the crevicular fluid and improved clinical adhesion levels (Berlutti et al., 2011). Several studies were conducted on *Staphylococcus* spp. 1,2,3,4,6-Penta-O-galloyl-β-D-glucopyranose, a plant-derived ingredient with iron-chelating properties commonly used in Chinese medicine, has shown biofilm inhibitory effects on *S. aureus* (Lin et al., 2012). The combination of deferiprone and the heme analog gallium protoporphyrin in combination with gentamicin or ciprofloxacin increased the biofilm inhibitory effects in an *S. aureus* colony biofilm model (Richter et al., 2017). Additionally, for coagulase-negative staphylococci, deferiprone showed enhanced antibacterial activity in combination with clindamycin, gentamicin, or vancomycin by disrupting biofilms (Coraça-Huber et al., 2018). Regarding fungi, deferiprone inhibited biofilm formation by *A. fumigatus*, whereas deferoxamine had the opposite effect, and promoted biofilm formation. The result of the promotion of biofilm formation by deferoxamine may be due to *A. fumigatus* acquiring more iron chelator complexes through a siderophore-like mechanism (Nazik et al., 2015). In addition, deferoxamine is used by zygomycetes and *A. fumigatus* as a siderophore to promote bacterial growth (Boelaert et al., 1993). Thus, depending on the combination of the type of microorganism and iron chelator, the microorganism has a tolerance mechanism and takes advantage of iron starvation. However, iron chelators have a different point of action than do antibacterial and antifungal agents, and thus, may carry less risk of inducing drug resistance in microorganisms. This is a major advantage of combining drugs with different mechanisms of action.

## Mechanisms of antimicrobial and anti-biofilm activity of iron chelators and antimicrobial-impregnated catheters

The iron chelator CP762 in combination with tetracyclines against *P. aeruginosa* showed synergistic effects. Among the tetracyclines, doxycycline showed particularly high synergism (Faure et al., 2021). CP762 is a hexadentate hydroxypyridinone iron chelator that has high affinity and selectivity for iron and does not utilize many of the bacterial iron siderophore receptors, making it unlikely to donate iron to pathogenic microbes (Piyamongkol et al., 2005; Zhou et al., 2011). Tetracycline binds to the 30S bacterial ribosome via a magnesium bridge (White and Cantor, 1971; Pioletti et al., 2001), but iron can inhibit this mechanism by binding to the magnesium binding site (Faure et al., 2021). Sequestration of iron by iron chelators may minimize iron binding to tetracycline, which would facilitate its complexation with low-affinity ions such as magnesium, which is necessary for binding to the bacterial ribosome (Faure et al., 2021).

## Conclusion

**Figure 1:**
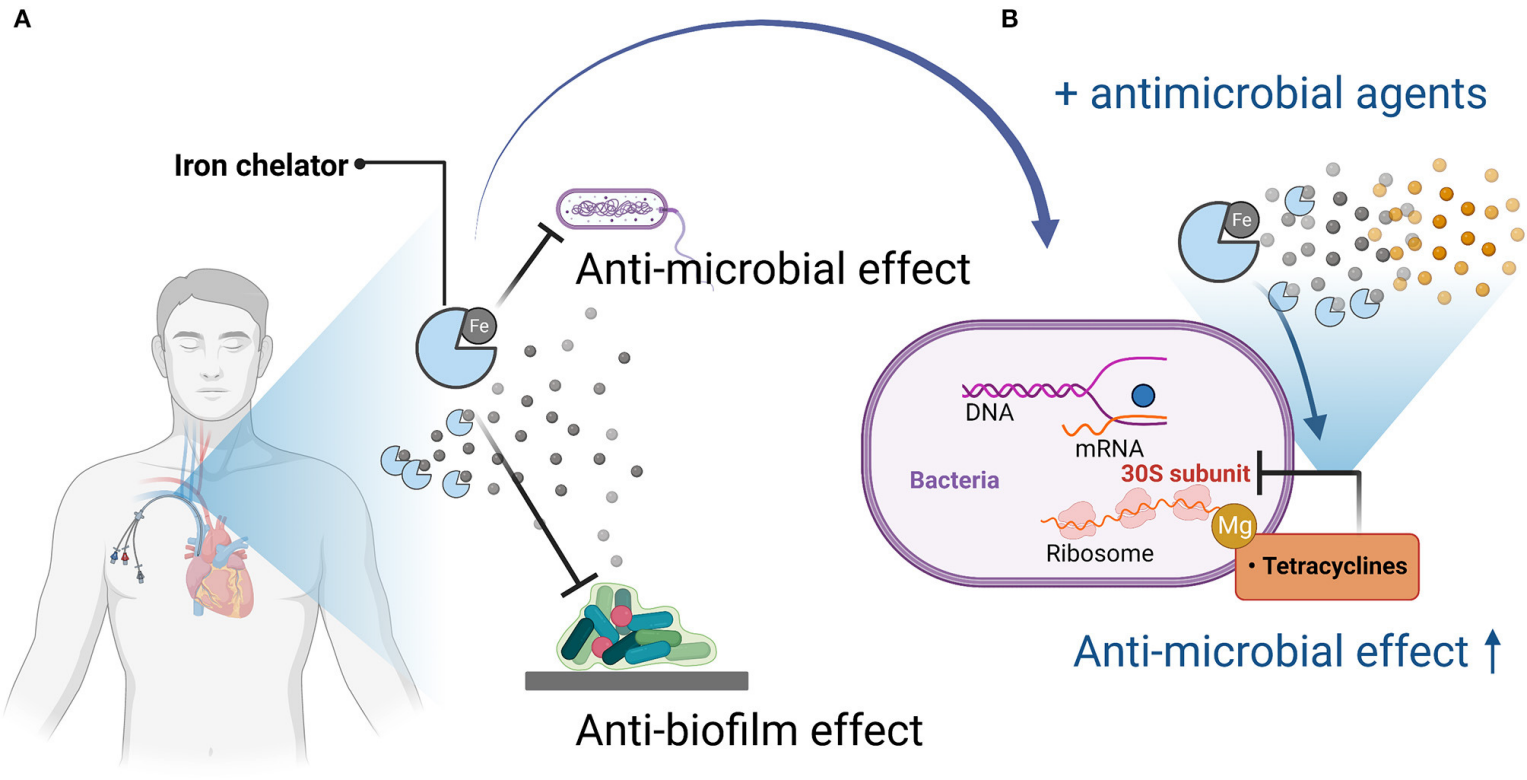
Mechanisms of antimicrobial and anti-biofilm activity of iron chelators and enhancement of antimicrobial activity when used in combination with antimicrobial agents. **(A)** Iron chelators exert antimicrobial and anti-biofilm activity by depriving pathogenic microorganisms of iron, which is necessary for their growth and proliferation and for biofilm formation. **(B)** When used in combination with an antimicrobial agent (tetracycline), tetracycline acts on the bacterial 30S ribosome via magnesium, but iron competes with magnesium, so iron chelation reduces the amount of iron bound to tetracycline, thereby promoting binding to magnesium, resulting in enhanced antimicrobial activity. Figures created with BioRender (https://biorender.com/).

Most iron chelators examined in the past have been effective in inhibiting biofilm formation by bacteria and fungi, and their antimicrobial activity has been shown to be enhanced when used in combination with antimicrobial agents (Figure 1, Table 1). Therefore, we believe that the inclusion of iron chelators in antimicrobial- or antifungal-impregnated catheters could help overcome microbial resistance mechanisms and create CVCs with a lower risk of CRIs. Further research on iron chelators and antimicrobial-impregnated catheters, and confirmation of their effects on reducing the incidence of CRIs in clinical trials, are warranted.

**Table 1:** Previous studies of antibacterial and anti-biofilm activity with antibacterial/antifungal agents and iron chelators.

**References**	**Type of study**	**Iron chelators**	**Adjuvant treatment**	**Pathogen**	**Relevant results/conclusion**
**Antibacterial activity**					
Luo et al. (2014)	*In vitro* and *in vivo* study	Deferasirox	Vancomycin	MRSA; VISA	Deferasirox enhanced vancomycin-mediated killing of MRSA and VISA *in vitro* (time-kill assays) and *in vivo* (*S. aureus* bacteremic mice) by a mechanism that appears to enhance vancomycin binding to the staphylococcal surface.
Faure et al. (2021)	*In vitro* study	CP762	Doxycycline; Minocycline; Oxytetracycline; Tetracycline; Tigecycline; Tobramycin	*P. aeruginosa*	Synergistic effects were observed between CP762 and all tetracyclines except minocycline against *P. aeruginosa* strains, while tobramycin showed no synergistic effects. In addition, the combination of doxycycline and CP762 significantly reduced cell viability in established biofilms.
Chan et al. (2020)	*In vitro* study	Deferasirox	Thiostrepton; Doxycycline	*P. aeruginosa*; *A. baumannii*	Fourteen compounds and one iron analog reported to have iron chelating activity were selected and tested for synergism with thiostrepton. The triple combination of thiostrepton, deferasirox, and doxycycline was the most effective against *P. aeruginosa* and *A. baumannii* isolates.
Ibrahim et al. (2010)	*In vitro* and *in vivo* study	Deferasirox	L-AMB	*A. fumigatus*	Deferasirox demonstrated *in vitro* fungicidal activity against *A. fumigatus* and prolonged survival in mice with invasive pulmonary aspergillosis. Deferasirox plus L-AMB synergistically improved survival and reduced pulmonary fungal burden compared to either agent alone.
Ibrahim et al. (2011)	*In vivo* study	Deferasirox	L-AMB; Micafungin	*R. oryzae*; *A. fumigatus*	Triple therapy with L-AMB, micafungin, and deferasirox improved survival and reduced tissue fungal burden in mice with mucormycosis, but to a lesser extent in aspergillosis.
Chayakulkeeree et al. (2020)	*In vitro* study	Deferoxamine; Deferasirox	AMB	*C. neoformans*; *C. gattii*	*C. neoformans* showed significant growth retardation when cultured in combination with AMB and deferoxamine, while *C. gattii* showed less growth retardation with deferoxamine plus AMB; no growth retardation of cryptococci was observed when deferasirox and AMB were used together.
Ibrahim et al. (2007)	*In vitro* and *in vivo* study	Deferasirox	L-AMB	*R. oryzae*	Deferasirox effectively chelates iron from *R. oryzae* and demonstrated *in vitro* killing activity against clinical isolates of Mucorales at concentrations well below clinically achievable serum levels. When used in combination with L-AMB, deferasirox synergistically improved survival and reduced tissue fungal burden.
Spellberg et al. (2012)	Randomized controlled trial	Deferasirox	L-AMB	*Rhizopus spp.; R. oryzae; R. microsporus; Cunninghamella spp*.	Twenty patients with proven or probable mucormycosis were randomized to treatment with L-AMB + deferasirox or L-AMB + placebo. Death at 30 days (45% vs. 11%, *P* = 0.1) and 90 days (82% vs. 22%, *P* = 0.01) were more frequent in the deferasirox group; global success (survival, clinical stability, radiographic improvement) at 30 and 90 days was 18% vs. 67% (*P* = 0.06) and 18% vs. 56% (*P* = 0.2).
Parquet et al. (2019)	*In vitro* and *in vivo* study	DIBI	Ciprofloxacin	*A. baumannii*	DIBI inhibited clinical *A. baumanii* isolates at MICs below those of typical antibiotics; low-dose nasal administration of DIBI after intranasal challenge with ciprofloxacin-resistant *A. baumanii* LAC-4 significantly reduced the bacterial burden in mice and DIBI also inhibited the spread of infection to the spleen. Given the ciprofloxacin resistance of LAC-4, treatment of infected mice with ciprofloxacin alone was ineffective, but treatment with DIBI greatly enhanced the therapeutic effect.
Savage et al. (2018)	*In vitro* and *in vivo* study	DIBI	Fluconazole	*C. albicans*	DIBI inhibited the growth of *C. albicans in vitro* at relatively low concentrations, and this inhibition was reversed by the addition of iron; the combination of DIBI and various azoles showed stronger growth inhibition than azoles alone, as shown in fluconazole-resistant *C. albicans*. In an experimental model of *C. albicans* vaginitis, DIBI in combination with fluconazole significantly improved clearance of infection in mice inoculated with fluconazole-sensitive strains.
**Anti-biofilm activity**					
Houshmandyar et al. (2021)	*In vitro* study	Deferiprone		*P. aeruginosa*	Deferiprone showed moderate activity against *P. aeruginosa* cells grown on plankton and was effective in inhibiting biofilm formation.
Mettrick et al. (2020)	*In vitro* study	HBED	Colistin	*P. aeruginosa*	HBED, a synthetic hexadentate iron chelator, inhibited the growth and biofilm formation of all clinical strains of *P. aeruginosa* under aerobic and anaerobic conditions; HBED in combination with colistin significantly enhanced the microcolony killing effect of colistin, resulting in almost complete biofilm removal.
Moreau-Marquis et al. (2009)	*In vitro* study	Deferoxamine; Deferasirox	Tobramycin	*P. aeruginosa*	Tobramycin in combination with deferoxamine or deferasirox reduced the *P. aeruginosa* biomass of established biofilms by approximately 90% and reduced viable bacteria by 7 log units; neither tobramycin, deferoxamine, nor deferasirox alone had such a significant effect. The combination of tobramycin and iron chelators also prevented biofilm formation on human respiratory cells.
Moreau-Marquis et al. (2015)	*In vitro* study	ALX-109 [combination of an investigational drug containing lactoferrin and hypothiocyanite (a bactericidal agent)]	Tobramycin; Aztreonam	*P. aeruginosa*	ALX-109 inhibited *P. aeruginosa* biofilm formation but did not affect established biofilms; ALX-109 enhanced the ability of tobramycin and aztreonam to inhibit *P. aeruginosa* biofilm formation and reduce established biofilms.
Moon et al. (2013)	*In vitro* study	Deferasirox		*P. intermedia*	Deferasirox exhibited potent antibacterial activity against *P. intermedia*, partially inhibited bacterial growth, and significantly prolonged bacterial doubling time. Deferasirox also significantly reduced the biofilm-forming activity and biofilm formation of *P. intermedia*. In the ATP bioluminescence assay, Deferasirox significantly reduced biofilm bioactivity.
Lin et al. (2012)	*In vitro* study	PGG		*S. aureus*	PGG may be a candidate for the development of anti-biofilm products for clinical use due to its anti-biofilm activity and low cytotoxicity. Impairment of *S. aureus* biofilm formation by PGG was shown to be due to iron chelation. Iron supplementation complemented the effect of PGG and restored biofilm formation.
Richter et al. (2017)	*In vitro* and *in vivo* study	Deferiprone	Gentamicin; Ciprofloxacin	*S. aureus*	The combination of deferiprone-GaPP significantly reduced bacterial load and increased survival of *S. aureus* small colony variant-infected *C. elegans* in an artificial wound model. When deferiprone-GaPP was combined with gentamicin or ciprofloxacin in a colony biofilm model, deferiprone-GaPP was able to enhance the activity of gentamicin or ciprofloxacin.
Coraça-Huber et al. (2018)	*In vitro* study	Deferiprone	Clindamycin; Gentamycin; Vancomycin	Coagulase-negative *staphylococci*	Deferiprone alone had only a moderate inhibitory effect on biofilm growth, but the combination of deferiprone and the respective antibiotics significantly reduced bacterial counts by 2–3 logs compared to the effect of the antibiotic alone. In addition, the combination of deferiprone and antibiotics showed severe to complete destruction of the biofilm, which was not seen when antibiotics were administered alone.
Nazik et al. (2015)	*In vitro* study	Deferoxamine; Deferiprone		*A. fumigatus*	Deferoxamine concentrations below 1,250 μM had no effect, while 2,500 μM increased biofilm formation or preform biofilms of *A. fumigatus*. 156 to 2,500 μM deferiprone inhibited biofilm formation in a dose-response manner. In preform biofilms, deferiprone above 625 to 1,250 μM showed an inhibitory effect compared to controls. The results of deferoxamine-induced enhancement of biofilm formation were attributed to *A. fumigatus* acquiring many iron chelator complexes through a siderophore-like mechanism.

A. baumannii, Acinetobacter baumannii; A. fumigatus, Aspergillus fumigatus; DIBI, denying iron to bacterial infections; GaPP, gallium protoporphyrin; HBED, N, N'-bis (2-hydroxybenzyl) ethylenediamine-N, N'-diacetic acid; C. albicans, Candida albicans; C. elegans, Caenorhabditis elegans; C. gattii, Cryptococcus gattii; C. neoformans, Cryptococcus neoformans; L-AMB, liposomal amphotericin B; MRSA, methicillin-resistant Staphylococcus aureus; P. aeruginosa, Pseudomonas aeruginosa; PGG, 1,2,3,4,6-penta-O-galloyl-b-D-glucopyranose; P. intermedia, Prevotella intermedia; R. microsporus, Rhizopus microsporus; R. oryzae, Rhizopus oryzae; S. aureus, Staphylococcus aureus; VISA, vancomycin intermediate Staphylococcus aureus.

## Author contributions

Conceptualization, methodology, and writing—original draft: KI and HI. Resources and data curation: KI. Writing—review and editing and visualization: KI, HT, YM, and HI. Supervision: HT, YM, and HI. All authors contributed to the article and approved the submitted version.
