# Supplementary material for: Potential additional effects of iron chelators on antimicrobial- impregnated central venous catheters
Source: Front Microbiol. 2023 Aug 7;14:1210747. doi: 10.3389/fmicb.2023.1210747 (PMC10442153; doi:10.3389/fmicb.2023.1210747)
Supplement: Supplementary file 1 [file Presentation_1.PPTX]

## Slide 1
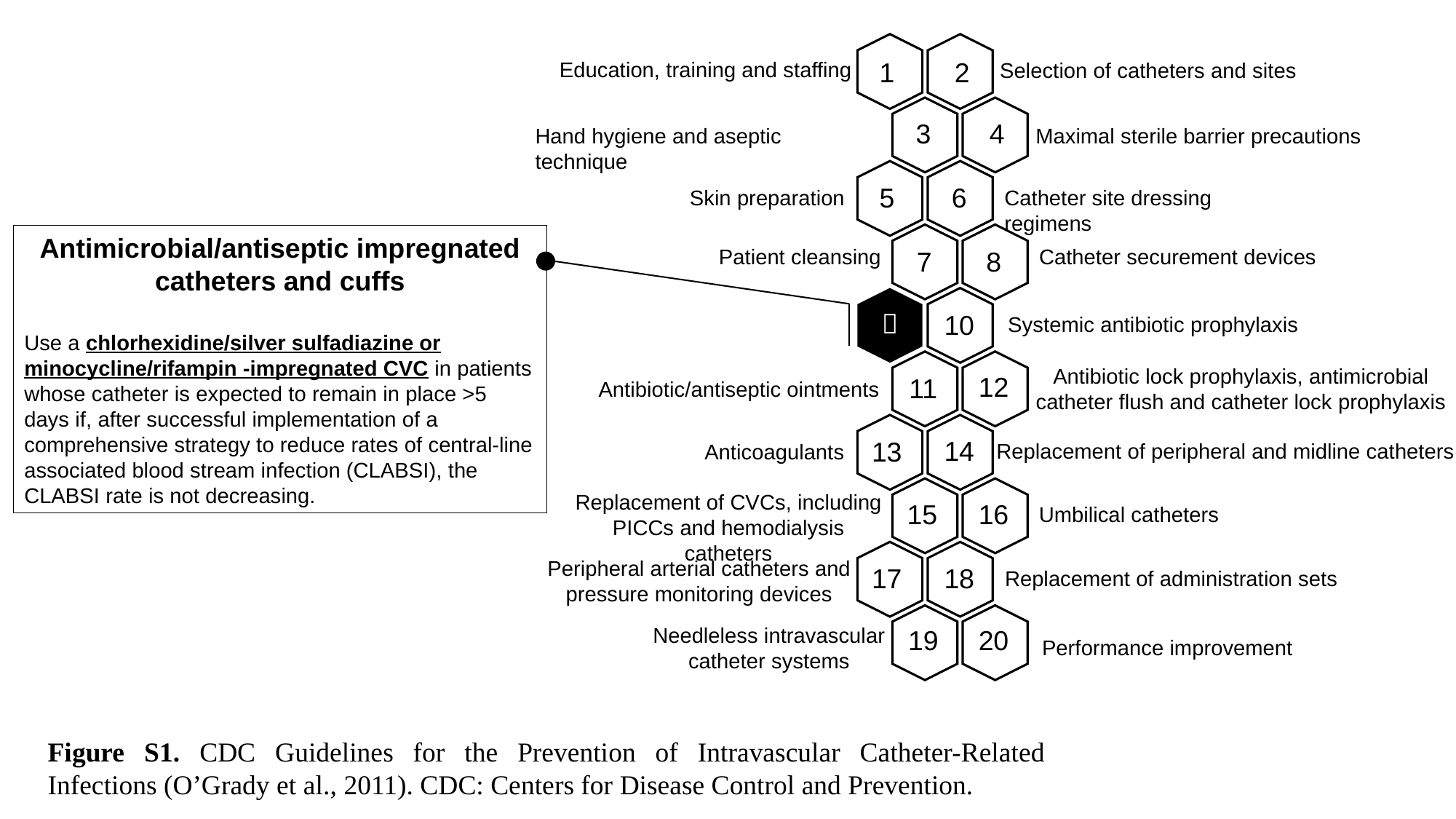

1
2
Education, training and staffing
Selection of catheters and sites
3
4
Maximal sterile barrier precautions
Hand hygiene and aseptic technique
5
6
Skin preparation
Catheter site dressing regimens
Antimicrobial/antiseptic impregnated catheters and cuffs
Use a chlorhexidine/silver sulfadiazine or minocycline/rifampin -impregnated CVC in patients whose catheter is expected to remain in place >5 days if, after successful implementation of a comprehensive strategy to reduce rates of central-line associated blood stream infection (CLABSI), the CLABSI rate is not decreasing.
Patient cleansing
Catheter securement devices
7
8
10
Systemic antibiotic prophylaxis
Antibiotic lock prophylaxis, antimicrobial catheter flush and catheter lock prophylaxis
12
11
Antibiotic/antiseptic ointments
14
13
Replacement of peripheral and midline catheters
Anticoagulants
Replacement of CVCs, including PICCs and hemodialysis catheters
15
16
Umbilical catheters
Peripheral arterial catheters and pressure monitoring devices
17
18
Replacement of administration sets
Needleless intravascular catheter systems
19
20
Performance improvement
Figure S1. CDC Guidelines for the Prevention of Intravascular Catheter-Related Infections (O’Grady et al., 2011). CDC: Centers for Disease Control and Prevention.
