# Supplementary material for: Potential additional effects of iron chelators on antimicrobial- impregnated central venous catheters
Source: Front Microbiol. 2023 Aug 7;14:1210747. doi: 10.3389/fmicb.2023.1210747 (PMC10442153; doi:10.3389/fmicb.2023.1210747)
Supplement: Supplementary file 2 [file Presentation_2.PPTX]

## Slide 1
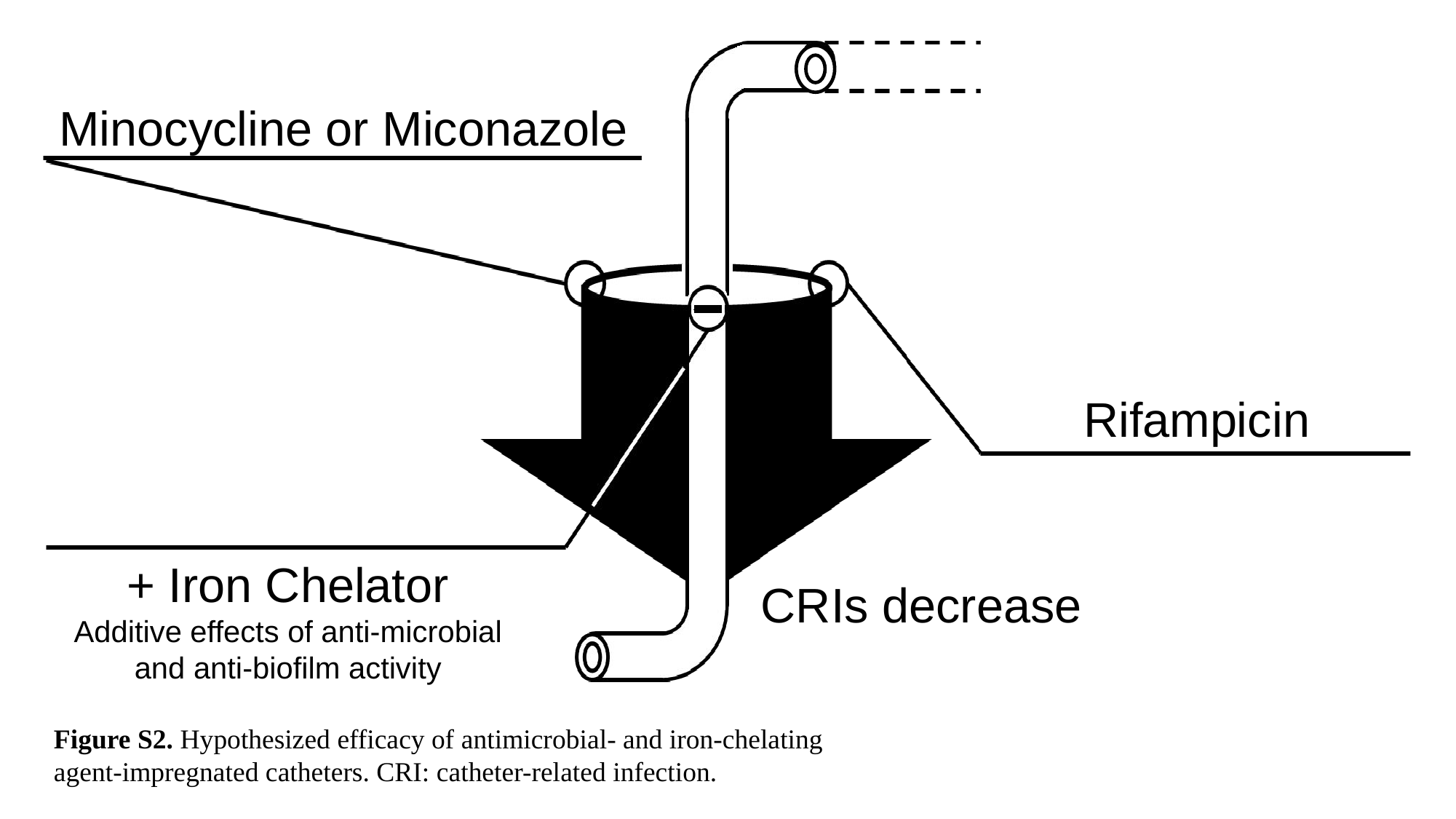

Minocycline or Miconazole
Rifampicin
+ Iron Chelator
Additive effects of anti-microbial and anti-biofilm activity
CRIs decrease
Figure S2. Hypothesized efficacy of antimicrobial- and iron-chelating agent-impregnated catheters. CRI: catheter-related infection.
